# Electron Transfer in Contact Electrification under Different Atmospheres Packaged inside TENG

**DOI:** 10.3390/ma16144970

**Published:** 2023-07-12

**Authors:** Yu Hou, Xuanli Dong, Wei Tang, Ding Li

**Affiliations:** 1 Center on Nanoenergy Research, School of Physical Science and Technology, Guangxi University, Nanning 530004, China; houyu@binn.cas.cn (Y.H.); dongxuanli@binn.cas.cn (X.D.); tangwei@binn.cas.cn (W.T.); 2 Beijing Key Laboratory of Micro-Nano Energy and Sensor, Beijing Institute of Nanoenergy and Nanosystems, Chinese Academy of Sciences, Beijing 101400, China; 3 School of Nanoscience and Engineering, University of Chinese Academy of Sciences, Beijing 100049, China

**Keywords:** package, atmosphere, contact electrification, TENG, electron transfer

## Abstract

Contact electrification (CE), a common physical phenomenon, is worth discussing. However, there are few reports on the influence of atmosphere on CE, or on the performance of triboelectric nanogenerators (TENG), based on CE by encapsulating gas inside. Here, we propose physical processes of electron transfer to interpret the impact of the gaseous atmosphere on CE. An atmosphere-filled triboelectric nanogenerator (AF-TENG) encapsulated five different gas-components of air based on the vertical contact separation mode was prepared. The sensitivity (1.02 V·N^−1^) and the power density (9.63 μW·m^−2^) of the oxygen-atmosphere-filled AF-TENG were 229.03% and 157.81% higher than these (0.31 V·N^−1^ and 3.84 μW·m^−2^) of the nitrogen-atmosphere-filled AF-TENG. As the oxygen atom possesses more atomic energy levels than other atoms, this could act as a “bridge” for more electrons to directly transfer between the two materials. The device package under different atmospheres could not only strengthen understanding of CE and improve the performance of TENG, but also be potentially applicable to prevent and control unnecessary damage caused by static electricity.

## 1. Introduction

Contact electrification (CE) or triboelectric electrification, a ubiquitous physical phenomenon, can be seen everywhere in daily life, for example, combing hair, undressing clothes, and slapping hands [1,2]. Recently, there have been many in-depth studies on the mechanism of CE [3,4,5,6,7]. Wang’s group [8] conducted a systematic investigation into the temperature-dependent real-time charge transfer in CE, using a triboelectric nanogenerator (TENG). They concluded that electron transfer is the predominant process for CE between metal and ceramic materials. Furthermore, they identified electron thermionic emission [9] as the primary inhibiting factor for CE at elevated temperatures. The electron transfer mechanism underlying CE was further validated at the nanoscale using Kelvin probe-force microscopy. Wang et al. demonstrated that electron transfer occurs in the repulsive region when two atoms are in close proximity to each other [10,11]. These observations led to the proposal of the overlapped electron-cloud model, which provides a comprehensive understanding of the electron transfer mechanism [12]. However, we live in a world full of atmosphere, there are relatively few studies on how the atmospheric conditions affect the electron transfer at the interfaces. Han et al. built a test system based on a piston-structured TENG, employing the contact–separation mode, to study short-circuit transfer charges and open-circuit voltages among three kinds of dielectric materials (polytetrafluoroethylene (PTFE), Polyimide film material (Kapton), polyethylene terephthalate (PET)) and in five pure gases (N_2_, O_2_, CO_2_, Ar, He) [13]. In this paper, the piston-structured TENG is driven by air pressure to generate contact separation, during which the gas is continuously exchanged between the interior and exterior. This can lead to electrons being consumed under this exchange. Lin et al.’s research on TENG, based on contact-separation mode in a closed vacuum chamber, studied transferred charge density among four kinds of dielectric materials (silicon dioxide (SiO_2_), aluminium nitride (AIN), polymethyl methacrylate (PMMA), also polyvinyl chloride (PVC)) and in four pure gases (N_2_, O_2_, Air, and Ar). The O_2_ molecule is found to shift the highest occupied surface state level (HOSL) of the dielectric to a lower energy level and make the dielectric more likely to be negatively charged [14]. This work solves the problem of gas exchange, but the gas studied is not comprehensive enough and the proposed mechanism is limited to the molecular scale, ignoring consideration of the atomic scale. Therefore, gaining further insights into the electron transfer between contacting materials in the presence of gases remains necessary.

Wang et al. created the TENG in 2012 [15]. It has been developed into five major application fields, including micro-nano energy [16,17,18,19,20,21,22,23,24], blue energy [25,26,27], self-powered sensors [23,28,29,30,31,32,33,34,35], high-voltage power supplies [36,37], and interface probes [3,38]. It is not only an idea meant to help study CE, but also excellent in practical applications. Liu et al. reduced the shielding effect of water through a novel structural design and increased the electrical output by 287% to collect ocean energy. Luo et al. prepared a wood-based triboelectric nanogenerator via special treatment of wood for self-powered sensing in athletic big-data analytics. The electrical output performance was enhanced by more than 70% compared with natural wood [39]. Kou et al. improve the electrical output performance of their friction layer material by 25% by doping the interior of porous PDMS with polyethylene terephthalate (FEP) particles of a specific size [30]. Chen et al. realized energy harvesting and sensing by using the anti-rotation structure TENG made of rabbit fur material [29]. More than ten times the electrical output based on the rabbit-fur material and a significantly elevated output current of 36.6% based on the anti-rotation structure TENG was observed relative to the conventional TENG [40]. Whether it is sensing or energy-harvesting devices based on TENG, previous studies mainly focused on the structural designs [41] or material choices [24,42,43,44], neglecting the packing gases inside TENGs. Therefore, investigating the performance of packaged TENGs under different atmospheres was also a significant direction for the future of TENG developments.

Here, we explored the impact of the gas atmosphere on CE through the atmosphere-filled TENG (AF-TENG) and propose a physical mechanism to interpret it. Besides the direct transfer of electrons between the two materials, it is also possible to transfer the electron through the energy levels of gas atoms as a “bridge”. We prepared the AF-TENG based on the vertical contact separation mode, and encapsulated five different gases related to air composition, including Ar, N_2_, air, CO_2_, and O_2_, inside the TENG through ecoflex. We found that the electrical properties of the oxygen-atmosphere-filled AF-TENG was the highest among them. As the O atom possesses more atomic energy levels than other atoms, this could act as a “bridge” to provide more channels for electron transfer. In applications, we applied an oxygen-atmosphere-filled AF-TENG to the fields of sensing and energy harvesting. It is demonstrated that both the sensitivity and the efficiency of energy harvesting can be enhanced by encapsulating oxygen inside the TENG. Overall, the package with different atmospheres was not only conducive to enhancing the performance of TENG-based sensing and energy harvesting, but also promoted a profound comprehension of CE. The underlying mechanism could be employed to more fields such as eliminating the damage to semiconductor devices caused by air breakdown and disasters caused by static electricity in life.

## 2. Materials and Methods

### 2.1. Experimental Materials

Kapton (DuPont 0.1 mm thick); PMMA (Jiangnan Hefeng Rubber and Plastic Products Factory 3 mm thick, Jiaxing, China); PTFE (Beijing Nuopude Company 0.1 mm thick, Beijing, China); ecoflex (BASF 0030); Gas cylinders (Beijing Hepu Beifen Gas Industry Co., Ltd. oxygen, nitrogen, carbon dioxide, and argon, Beijing, China); printed circuit board (PCB, JustFit 14 mm × 29 mm × 0.435 mm and 19 mm × 29 mm × 0.435 mm); silicone adhesive (Xinzhanxiang Company XZX-735, Beijing, China).

### 2.2. Fabrication of AF-TENG

**Figure 1:**
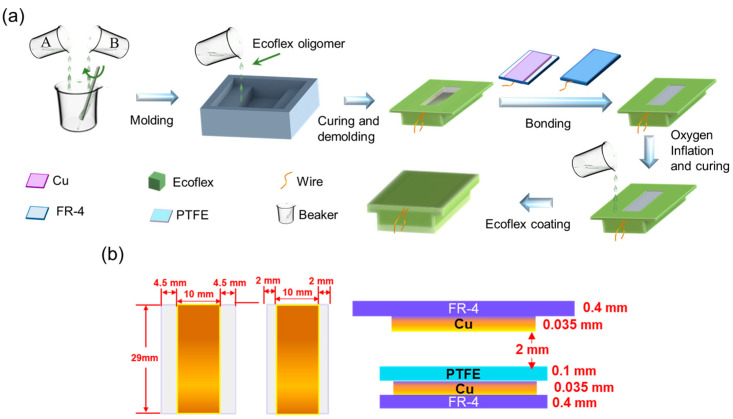
Materials and fabrication of AF-TENG. (**a**) Schematic illustration of the fabrication processes of AF-TENG. (**b**) The sizes and the thickness diagram of PTFE film and customized PCBs made of Cu and FR-4 plate.

The fabrication processes of the AF-TENG are depicted in Figure 1a. Initially, we mixed equal amounts of ecoflex glue A and ecoflex glue B into the container and stirred well. Then, we placed the uncured ecoflex in a vacuum chamber for five minutes to remove internal air bubbles. Next, ecoflex oligomer was poured into the mold and cured for six hours. After demolding, a cured T-shaped ecoflex shell with a hole (the space for deformation and atmosphere filling) for the AF-TENG was obtained. Next, we attached 100 μm-thick PTFE film to the lower customized PCB plate and soldered the wires to the pads of the PCB. The detailed size of the PCBs is shown in Figure 1b. The PCBs are bonded to the upper and lower inner surfaces of the ecoflex shell via the inflation of different gases. Then, the ecoflex shell was sealed with glue and cured for six hours. Finally, the device was coated with an ecoflex oligomer on its surface and cured for another six hours.

### 2.3. Inflation Method of AF-TENG

First, we applied silicone adhesive to the interface between the PCB and the ecoflex shell via a hole, and let the adhesive stand for an hour to make the silicone adhesive semi-dry to ensure that there would be no leakage after inflation. Then, we inserted a gas pipeline into the interface (leaving an exhaust port) and connected the other end of the gas pipeline to the gas cylinder to inflate the interior of the AF-TENG. After one minute, we quickly pressed the interface and replenished the silicone adhesive outside AT-TENG again to ensure the seal. Finally, after six hours of curing at room temperature, the gas encapsulation was completed.

### 2.4. Characterization and Measurement of AF-TENG

**Figure 2:**
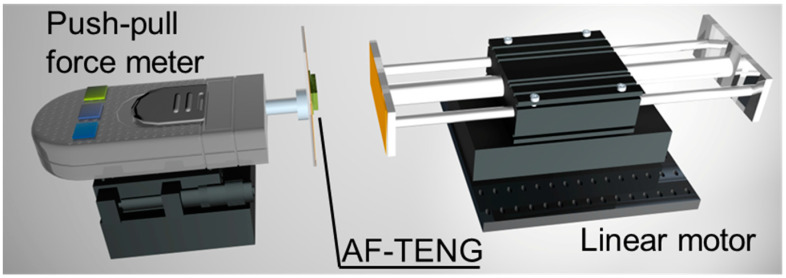
Test method for AF-TENG output performance. The AF-TENG is attached to a push-pull force meter and subjected to regular pressure.

The electrical outputs of the AF-TENG were obtained using the Keithley 6514 electrometer system. The normal force of the AF-TENG is controlled by a linear motor (LinMot E1100) with the Kapton sticker. The normal force of the AF-TENG was tested using a digital push-pull force gauge (Handpi SH-10N). Figure 2 illustrates the test method of the AF-TENG. The electrical outputs of AF-TENG were obtained via a linear motor that applies a normal force to the AF-TENG and the dynamometer that is used to test the force of the AF-TENG. A PMMA board with Kapton attached to the surface of the linear motor was bonded. Another PMMA board with Kapton attached to the surface of the dynamometer was bonded, and AF-TENG was pasted onto it. In this way, the external electrostatic induction could hardly affect the electrical output of AF-TENG. The surface potential distribution of the PTFE is tested using the Trek 370TR electrostatic voltmeter. We divided the PTFE surface of 14 mm × 29 mm into 406 grids of 1 mm^2^. We tested the surface potentials of each grid separately, recorded them, and processed them using Origin 2018. In order to measure the current and power densities, we obtained the short-circuit charge (*Q*_sc_), the open-circuit voltage (*V*_oc_), and the short-circuit current (*I*_sc_) by connecting loads with different resistances in series with TENG and tested them with a 6514 electrometer. Then we calculated the output power using P = I^2^R. Finally, the obtained data were processed and plotted using Origin 2018.

## 3. Results

### 3.1. Structure of AF-TENG

**Figure 3:**
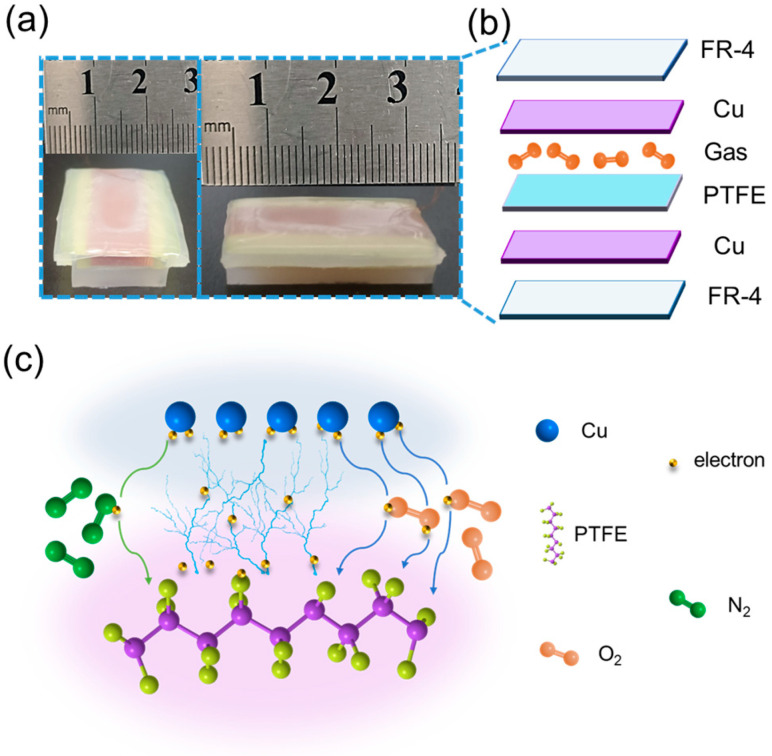
Schematic diagrams of the structure of AF-TENG. (**a**) Optical photographs of AF-TENG. (**b**) Schematic structure diagram of AF-TENG. (**c**) Schematic diagram of mechanism of contact electrification processes inside AF-TENG.

We demonstrated the structure and electron-transfer processes of the AF-TENG (Figure 3). The photograph of the AF-TENG and its schematic structure are shown in Figure 3a and Figure 3b, respectively. The AF-TENG comprised an upper customized PCB made of a Cu-top electrode and fiberglass board (FR-4), PTFE film, and a lower customized PCB made of a Cu-bottom electrode and another FR-4. The gas inside the AF-TENG and two PCB boards were encapsulated with an ecoflex shell (Figure 1a). The detailed fabrication processes of the AF-TENG are presented in of materials and methods section. The completed AF-TENG was 20 mm × 30 mm × 5 mm.

### 3.2. Proposed Mechanism

Figure 3c illustrates the overview of physical processes of electron transfer for interpreting the impact of the gases at CE. When Cu and PTFE are in contact with each other, the electrons will be directly transferred from Cu to the surface of PTFE since their electron clouds are overlapped. It is also possible that more electrons could be transferred when the gas atmosphere exists, which provides “bridges” for electrons, promoting the transfer of more electrons. In particular, O atoms have more energy levels than N atoms have, so O_2_ provides more “bridges” than N_2_ for electron transfer.

### 3.3. Experimental Results

#### 3.3.1. Principle of AF-TENG

**Figure 4:**
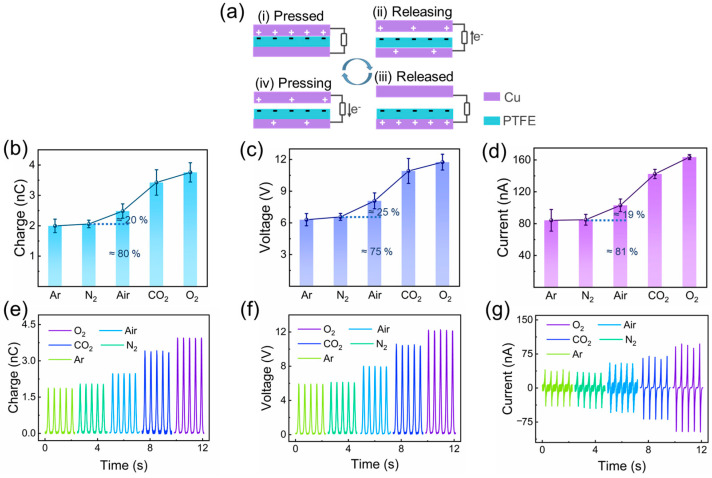
Schematic diagram of working principle and the effects of different atmospheres on the electrical output of the AF-TENG. (**a**) Working principle of the AF-TENG. (**b**–**d**) Evolution of short-circuit charge, open-circuit voltage, and short-circuit current of AF-TENG under various atmospheres (Ar, N_2_, Air, CO_2_, O_2_). (**e**–**g**) Electrical output signals of AF-TENG under various atmospheres (Ar, N_2_, Air, CO_2_, O_2_).

The test method and working principle of the AF-TENG, operating in vertical contact separation mode, are illustrated in Figure 2 and Figure 4a. The electrical outputs of AF-TENG are obtained through a linear motor that applies a normal force to the AF-TENG, which is bonded to the dynamometer (Figure 2). The detailed test method of the AF-TENG is demonstrated in the materials and methods section. Initially, when the top Cu electrode contacts the bottom PTFE, negative charges are induced on the PTFE surface, and the charges on the top Cu electrode, having opposite polarities, are compensated for by the bottom Cu electrode (Figure 4(ai)). This is attributed to the strong electronegativity of PTFE and the interaction between triboelectrification and electrostatic induction. When the top Cu electrode separates from the bottom PTFE, the current drifts from the top Cu electrode to the bottom Cu electrode, as sown in Figure 4(aii–iii). Subsequently, the current drifts from the bottom Cu electrode to the top Cu electrode when the top Cu electrode contacts the bottom PTFE are shown in Figure 4(aiii–iv). The charges on the electrodes revert to their original state when the top electrode returns to its initial position.

#### 3.3.2. Electrical Output of AF-TENG

**Figure 5:**
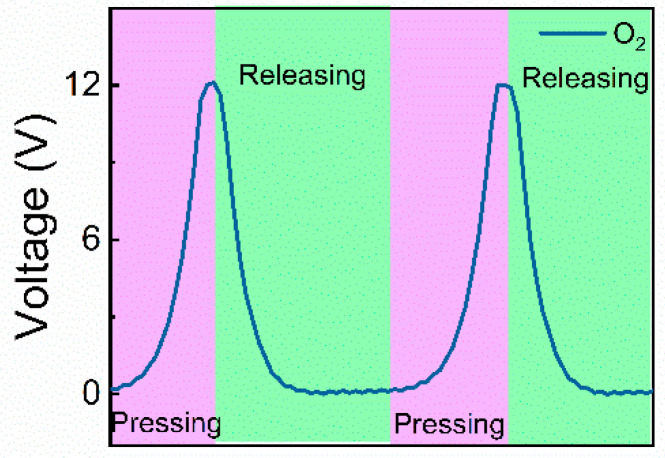
Changes of voltage signal corresponding to the processing of pressing and releasing of O_2_-atmosphere-filled AF-TENG under a 5.6 N normal force.

*Q*_s_, *V*_oc_, and *I*_sc_ of the AF-TENG filled with argon (Ar), nitrogen (N_2_), air, carbon dioxide (CO_2_), and oxygen (O_2_) atmospheres under 5.6 N normal force are obtained (Figure 4b–g). These gases are chosen as they are the four main components of air. The results showed the electrical outputs of the AF-TENG filled with pure Ar, and N_2_ atmospheres were *Q*_sc_ of about 2 nC, *V*_oc_ of about 6.2 V, and *I*_sc_ of about 82 nA, which were lower than other situations. The one with the pure O_2_ atmosphere was *Q*_sc_ of about 3.75 nC, *V*_oc_ of about 12 V, and *I*_sc_ of about 160 nA, which were the best among five gas atmospheres. The one with the pure CO_2_ atmosphere was *Q*_sc_ of about 3.5 nC, *V*_oc_ of about 11 V, and *I*_sc_ of about 145 nA, which were the second followed by the one with air. Specifically, about 80%, 75%, and 81% of the *Q*_sc_, the *V*_oc_, and the *I*_sc_ of the air-atmosphere-filled TENG, which were about 2.5 nC, about 8 V, and about 105 nA, are just the *Q*_sc_, the *V*_oc_, and the *I*_sc_ of the N_2_-atmosphere-filled TENG, which were close to the N_2_ content of air (~78%). Figure 4e–g shows the typical electrical output signals of AF-TENG filled with different gas atmospheres. The enlarged normal force-signal changes corresponding to the pressing and the releasing of O_2_-atmosphere-filled AF-TENG are shown in Figure 5.

#### 3.3.3. Surface Potential Distributions of the PTFE Film under Different Atmospheres

**Figure 6:**
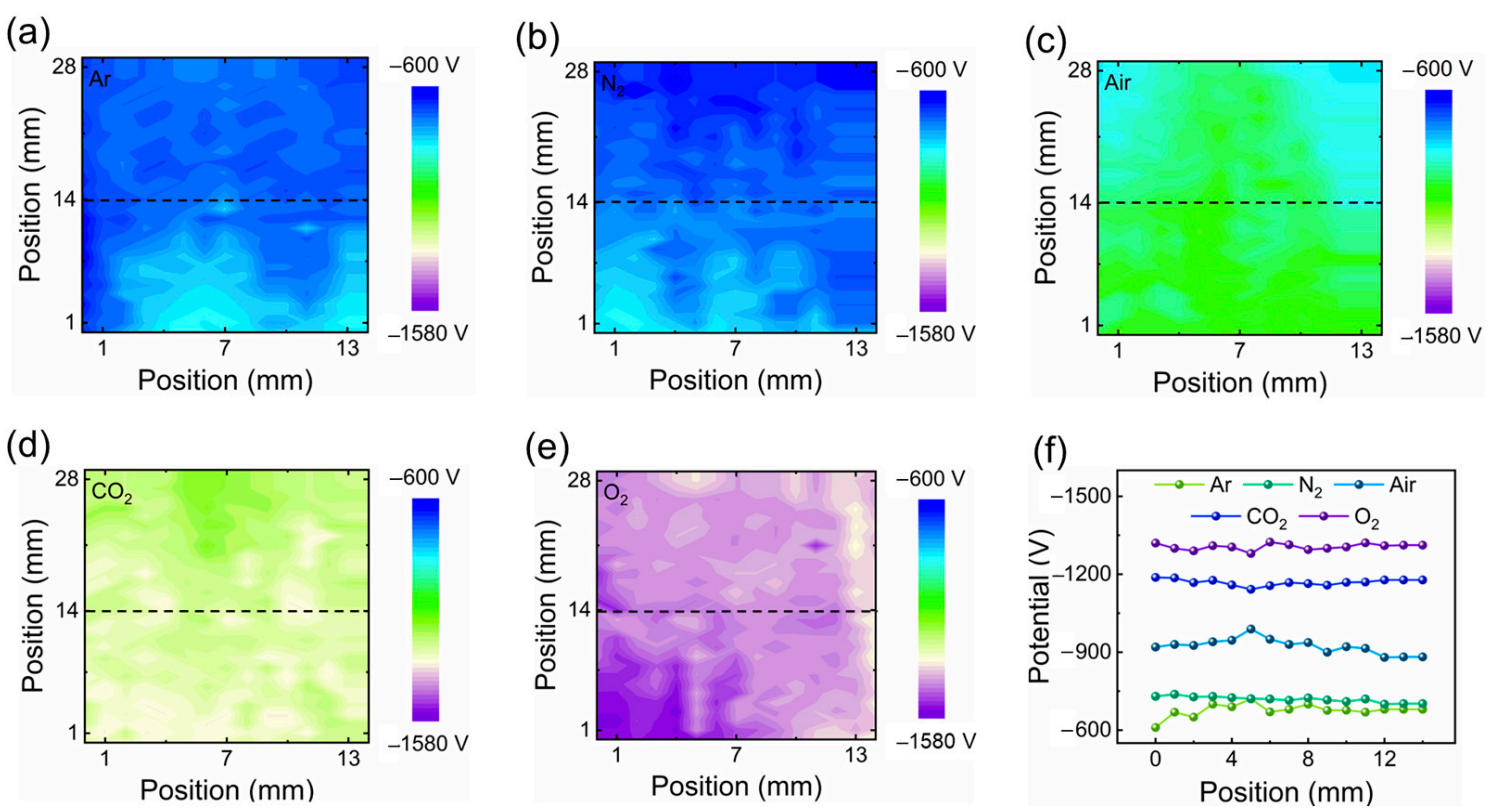
The surface potential distributions of PTFE films. (**a**–**e**) The recorded surface potential of PTFE films after they were contacted by Cu under various atmospheres (Ar, N_2_, Air, CO_2_, O_2_). (**f**) The profiles of potential on the PTFE films at various atmospheres (Ar, N_2_, Air, CO_2_, O_2_) extracted from black dotted lines of *x*-axis direction.

In order to prove that the differences of AF-TENG with different gas atmospheres on previous tests were directly from electron transfer between the Cu electrode and the PTFE film, the surface potential distributions of the PTFE film under different atmospheres were tested (Figure 6). At first, the AF-TENGs are continuously contacted and separated by a normal force of 5.6 N until reaching saturation. After that the PTFEs were taken out from AF-TENGs and the surface potential distribution was tested immediately (in the *x*-axis direction, the record range was selected from 0 to 14 mm; in *y*-axis direction, the record range was selected from 0 to 29 mm). Considering that the electrons on the dielectric material are not easily dissipated, we could obtain a relatively reliable result of the potential distribution. The surface potential distributions of the AF-TENG filled with pure Ar, N_2_, air, CO_2_, and O_2_ atmospheres are shown under the same scaling range in Figure 6a–e, respectively. The more the electrons transferred, the more negative potential there was. The results show the absolute values of the surface average potentials of the AF-TENG filled with pure Ar, and N_2_ atmospheres were lower than other situations. The one with the pure O_2_ atmosphere was the best among five gas atmospheres. The one with the pure CO_2_ atmosphere was second, followed by the one with air. Figure 6f summarizes a line scan of the surface potential distribution with different atmosphere denoted by the black-dotted line. About 78% of the PTFE surface average potential of the air-atmosphere-filled TENG, which is about −900 V, was just one of the N_2_-atmosphere-filled AF-TENG, which is about −700 V. These results are consistent with the previous experimental results on the Q_sc_, the V_oc_, and the I_sc_. They demonstrate that the differences of AF-TENG with different gas atmospheres were directly due to electron transfer differences between the Cu electrode and the PTFE film under different gas atmospheres.

## 4. Discussion

### 4.1. Physical Processes of Electron Transfer

**Figure 7:**
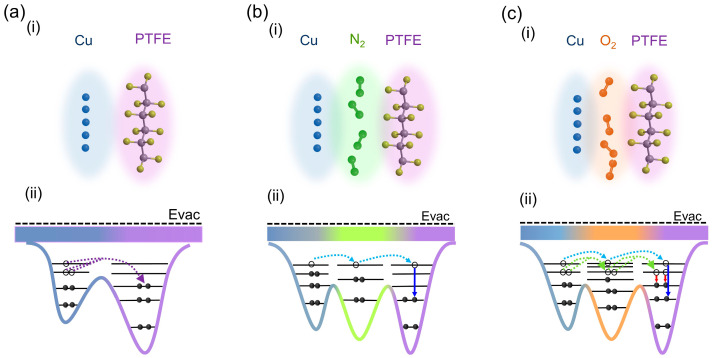
Schematic diagrams of the proposed physical processes of electron transfer between Cu and PTFE. (**ai**,**bi**,**ci**) Molecular schematic diagram and (**aii**,**bii**,**cii**) corresponding energy level diagram under (**a**) no atmosphere, (**b**) nitrogen atmosphere, and (**c**) oxygen atmosphere, respectively.

Based on previous experimental results, we propose physical processes using the energy-level diagram as shown in Figure 7. Initially, Cu electrodes do not contact the PTFE, and there is no electron transfer between them. When the surface of the Cu electrode contacts PTFE, the electron clouds of the atoms at the surface of the two materials overlap (Figure 7(ai)) and the electron could transfer between them based on the overlapped electron-cloud model (Figure 7(aii)) [12]. Besides the direct electron transfer between two materials, the atoms or molecules in the atmosphere might also play a role in electron transfer during contact electrification [14]. Since N_2_ and O_2_ are the main components of air, we would like to discuss the possible physical processes of electron transfer under N_2_ and O_2_ atmospheres according to our experimental results. In a pure N_2_ atmosphere as shown in Figure 7b, some of the electrons transferred from Cu to PTFE via N_2_ act as a “bridge”. In this process, electrons tunnel from the energy level of Cu to the energy levels of the O atoms, and then tunnel from the energy levels of the O atoms to the energy levels of PTFE. Electrons tunneling to a higher energy level of PTFE transition to a lower energy level inside the atom. While in a pure O_2_ atmosphere, some of the electrons transferred from Cu to PTFE via O_2_ act as a “bridge” (Figure 7c). Considering that the energy levels of the O atom are more numerous than that those of the N atom, it is possible that more electrons could transfer from Cu to PTFE through these energy levels as bridges. This is because electrons have more opportunities to tunnel between energy levels of Cu, O, and PTFE. The green and blue dotted lines represent tunneling at different energy levels, respectively. The blue and red solid lines represent electronic transitions between different energy levels, respectively. The energy levels of O atoms are more than those of N atoms, based on the number of atomic spectral lines in the atomic spectrum database. More atomic spectral lines corresponds to more electronic transitions with more energy levels. In the case of atoms with the same energy levels, for example, both CO_2_ and O_2_ have two O atoms, O_2_ has two unpaired electrons, exhibiting its paramagnetism, while CO_2_ does not have unpaired electrons [45]. Unpaired electrons are freer and easier to transfer compared to paired electrons. Therefore, the performance of AF-TENG filled with an O_2_ atmosphere is slightly higher than that of AF-TENG filled with a CO_2_ atmosphere. In addition, it is also possible that O_2_ plays a role as an electron acceptor; however, N_2_ is less electronegative than O_2_ and is generally not an efficient electron acceptor. The experiments show that electrical outputs of the AF-TENG filled with an atmosphere of pure O_2_ are the best among the five typical atmosphere situations. This means that more electrons are transferred in the O_2_ atmosphere compared with those in the N_2_ atmosphere and in the air. From our previous experimental data, about 78% of the electrical outputs and the PTFE surface average potential of the air-filled atmosphere AF-TENG equal those of the N_2_-atmosphere-filled AF-TENG. This is consistent with about 78% of air being composed of N_2_ and about 21% of air composed of O_2_. So we confirm the proposed physical processes.

### 4.2. Practical Application Potential of AF-TENG

Altought the enhancements of the performance of TENG mainly focus on the structure design and material improvements, the package of TENG is also important, which is usually neglected. In our above experiments, we shed light on the improvement of TENG via the package. Given the same materials choices and the same structural designs, different packages might lead to different results. Most previous studies focused on the TENG with structural designs [46] or materials choices [47] in an air atmosphere, ignoring the method to enhance the performance of TENG by encapsulating the gas atmosphere. Therefore, in order to improve the sensing and energy-harvesting performance of these TENGs, we selected the oxygen-atmosphere-filled AF-TENG with the best electrical outputs to improve the air-atmosphere-filled AF-TENG. Next, we tested the sensitivity and power density of AF-TENG in air and in O_2_ atmospheres, respectively.

**Figure 8:**
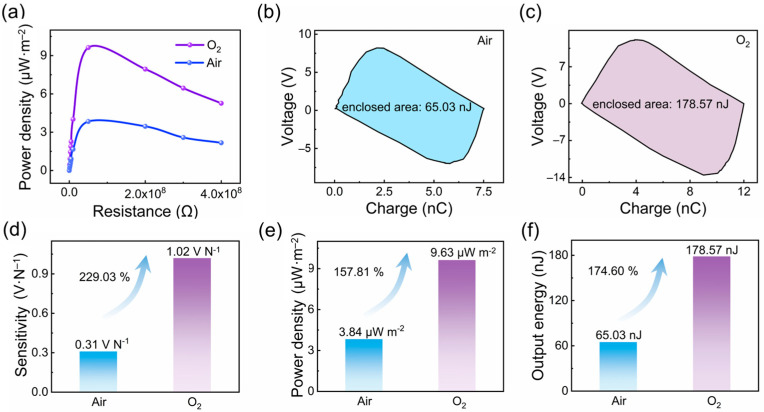
Comparasion of the performance of AF-TENG filled with O_2_ and air atmospheres. (**a**) The relationship between resistance and powder density, and (**b**,**c**) VQ curves of AF-TENG filled with O_2_ and air atmospheres, respectively. Comparison of (**d**) the sensitivity, (**e**) the powder density, and (**f**) output energy between AF-TENG filled with O_2_ and air atmospheres.

Figure S1 shows the voltage sensitivities of AF-TENG with O_2_ and air atomsphere under different forces, respectively, which are the linear-fitting results based on the experimental data. Although both force-electricity relationships have very good linearity, the sensitivity of the oxygen-atmosphere-filled AF-TENG (1.02 V·N^−1^) was more than three times higher than that of the air-atmosphere-filled AF-TENG (0.31 V·N^−1^). The sensitivity of the oxygen-atmosphere-filled AF-TENG increased by 229.03% more than that of the air-atmosphere-filled TENG, as shown in Figure 8d. Therefore, the sensitivity of TENG-based sensing devices can be greatly improved by encapsulating O_2_ inside the TENG during package.

Figure 7a and Figure S2 show the current density and the power density of AF-TENG with air and O_2_ atomsphere under external resistors of different resistances, respectively. It can be seen that the peak power density of the oxygen-atmosphere-filled AF-TENG (9.63 µW·m^−2^) was significantly higher than that of the air-atmosphere-filled AF-TENG (3.84 µW·m^−2^). The peak power density of the oxygen-atmosphere-filled AF-TENG was 157.81% higher than that of the air-atmosphere-filled AF-TENG under the same structure, as shown in Figure 8e.

The VQ curves for the two typical gases (O_2_ and Air) were tested, as shown Figure 8b,c. As a result, the enclosed area of the AF-TENG filled with an O_2_ atmosphere (178.57 nJ) was higher than that of the AF-TENG filled with an air atmosphere (65.03 nJ). The maximum output energy of the oxygen-atmosphere-filled AF-TENG was 174.60% higher than that of the air-atmosphere-filled AF-TENG given the same structure, as shown in Figure 8e. Therefore, the power density of TENG-based energy-harvesting devices can be greatly improved via a method of encapsulating O_2_ inside the TENG during the package.

## 5. Conclusions

In this paper, physical processes are proposed to interpret the impact of gas atmosphere on CE. Besides the direct transfer of electrons between the two materials, it is possible to understand the electron transfer through the energy levels of gas atoms as a “bridge”. An atmosphere-filled TENG (AF-TENG) of the vertical contact separation mode, based on the coupling between triboelectrification and electrostatic induction, is fabricated. It encapsulates different gas atmospheres, including Ar, N_2_, CO_2_, air, and O_2_, inside it via ecoflex shells. The experiments show that the electrical outputs and the potential on PTFE film of the oxygen-atmosphere-filled AF-TENG were the highest among the five typical atmosphere situations. As the number of energy levels of the O atom is more than that of the other atoms, it is possible that more electrons could transfer through these energy levels as bridges. Furthermore, we apply the oxygen-atmosphere-filled AF-TENG to the fields of sensing and energy harvesting. The sensitivity, the peak power density, and the maximum output energy can be enhanced by 229.03%, 157.81%, and 174.60% compared to that of the air-atmosphere-filled TENG. In addition to improving device performance, adjusting the atmosphere may potentially also be used to reduce the ability of CE for decreasing unnecessary static electricity generated in life by changing the atmosphere of the gas. For example, we can encapsulate nitrogen or argon atmospheres into the semiconductor device to reduce the electrostatic breakdown of the device caused by CE for avoiding device damage. We can also pump nitrogen or argon into the unmanned automation workshop to reduce the possibility of unnecessary disasters caused by static electricity in workshops with explosive dust. Overall, the package under different atmospheres is not only conducive to strengthening the performance of TENG, but also promotes a deep understanding of CE. In the future, it even has the potential to prevent and control unnecessary damage to life and to benefit mankind.

## Data Availability

The data presented in this study can be obtained from the corresponding author upon reasonable request.

## Supplementary Materials

The following supporting information can be downloaded at: https://www.mdpi.com/article/10.3390/ma16144970/s1, Figure S1: the sizes and the thickness diagram of PTFE film and customized PCBs made of Cu and FR-4; Figure S2: changes of voltage signal corresponding to the pressing and the releasing processing under 5.6 N normal force.
