# Supplementary material for: Electron Transfer in Contact Electrification under Different Atmospheres Packaged inside TENG
Source: Materials (Basel). 2023 Jul 12;16(14):4970. doi: 10.3390/ma16144970 (PMC10382056; doi:10.3390/ma16144970)
Supplement: Supplementary file 1 [file materials-16-04970-s001.zip › materials-2464191-supplementary.pdf]

Supplementary Materials

Electron Transfer in Contact Electrification under Different Atmospheres Packaged Inside TENG

Yu Hou [1,2,†], Xuanli Dong [1,2,†], Wei Tang [1,2,3] and Ding Li [1,2,3,*]

[1] Center on Nanoenergy Research, School of Physical Science and Technology, Guangxi University, Nanning 530004, China
[2] Beijing Key Laboratory of Micro-Nano Energy and Sensor, Beijing Institute of Nanoenergy and Nanosystems, Chinese Academy of Sciences, Beijing 101400, China
[3] School of Nanoscience and Engineering, University of Chinese Academy of Sciences, Beijing 100049, China
* Correspondence: liding@binn.cas.cn
† These authors contributed equally to this work.

Supplementary Figures S1 and S2

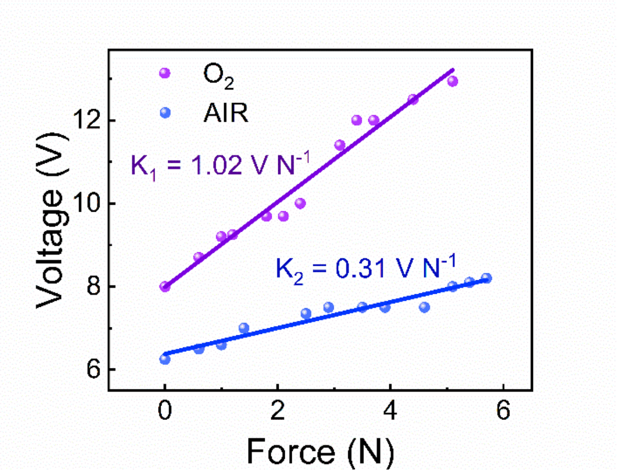

Figure S1. The relationship of force and voltage of AF-TENG filled with O_2 and air atmospheres.

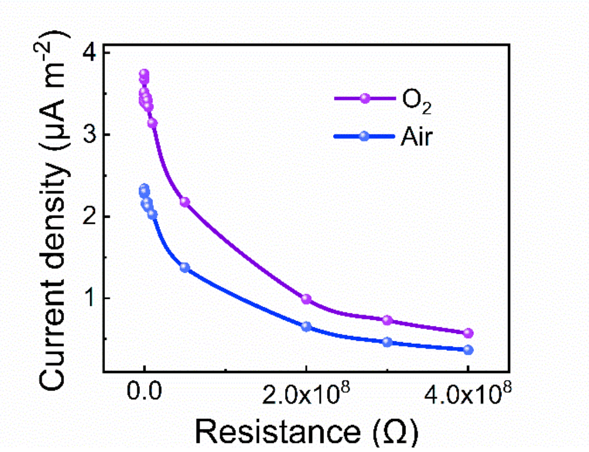

Figure S2. The relationship of resistance and current density of AF-TENG filled with O_2 and air atmospheres
